# Reductionist and system approaches to study the role of infection in preterm labor and delivery

**DOI:** 10.1186/1471-2393-7-S1-S9

**Published:** 2007-06-01

**Authors:** Piotr Laudanski, Piotr Pierzynski, Tadeusz Laudanski

**Affiliations:** 1 Department of Pathophysiology of Pregnancy, ul. Marii Sklodowskiej-Curie 24a 15-243 Bialystok, Medical University of Bialystok, Poland

## Abstract

A substantial number of patients with preterm labor and delivery do not show clinical signs of infection, however, it is the subclinical form which is the main causative factor and often results in premature delivery. The hitherto commonly applied methods of inflammation detection are based either on potentially hazardous amniocentesis or still insufficient inflammation-related protein measurement in the serum or other biological fluids.

The advent of new "omics" technologies has led to a paradigm-shift in experimental approach which tends to primarily generate rather than form hypotheses. This has resulted in a surge of wealth of data composed of sets of individual or clusters of new genes and proteins that can be of potential importance as new markers of inflammation leading to preterm labor. It is hoped that as a result of those new methodologies the overall perception of medical research and practice would gradually change from reductionist to systems approach. Despite several successes of reductionism in the diagnosis and treatment of preterm labor it seems that system-based methodology would contribute to a more favorable personalized rather than one-for-all patient assistance. In this review we present the current knowledge on this new attractive field of medical studies with emphasis on early detection of infection related with preterm labor.

## Introduction

Preterm birth is the ultimate result of several different pathways that culminate in the initiation of labor before 37 weeks' gestation. The rate of premature births ranges between 3,5% (Holland) and 34% (India) and is strongly dependant on mother's socioeconomic status and/or familial factors [1-3]. Significant disparities in preterm birth rates exist between racial and ethnic groups according to most recent Institute of Medicine report on preterm birth [4]. It may be due to a number of factors such as: nutrition, cigarette smoking, substance use or abuse, work and physical activity, prenatal care or infection, however, it is most feasible that cultural and sociological factors are mainly responsible for the increase in preterm birth rates in the past decade. The trend toward having children later in life may be contributing because older women are more likely to undergo infertility treatment with increased chance of multiple pregnancy. Although there is no proof that the rate of infection has been recently augmented it has relatively well-established molecular physiology and has been widely accepted as most probable cause of preterm birth [5].

## Infection and premature birth

Infection, (particularly unrecognised/subclinical infection *in utero*) is considered an aetiological factor in around 50% of cases. It is more likely to be associated with deliveries that occur before 30 weeks, as shown by histological examination of the fetal membranes at delivery, and is relatively rare in late preterm deliveries (at 34 to 36 weeks). It was shown that up to 80% of women who deliver before 30 weeks gestation have evidence of bacterial infection in the amniotic fluid (MIAC – microbial invasion of the amniotic cavity) and/or membranes compared with only 30% at 37 weeks gestation [6].

Bacterial infection within the uterus can occur within the choriodecidual space and most commonly affects fetal membranes. Resulting chorioamnionitis is usually documented by histological findings or culture. Merely 10% of cases manifest clinically during pregnancy by typical signs and symptoms i.e. one temperature elevation of >38°C, combined with at least two of the following signs: maternal or fetal tachycardia, uterine tenderness greater than expected, foul-smelling vaginal discharge, and white blood cell count of >18,000.

The uterus can be infected by different types of microorganisms via number of routes and haematogenous spread through the placenta, or ascending from vagina are the most common causes. Among vaginal infections the most important are: bacterial vaginosis, chlamydial and trichomonas infection as well as aerobic vaginitis [7].

There is accumulating evidence suggesting that it is chronic rather than acute inflammatory reaction which is associated with the development of chorioamnionitis leading to preterm delivery [8]. Organisms frequently cultured following preterm delivery include genital *Mycoplasma *species, in particular *Ureaplasma urealyticum*, however other microorganisms are also found, such as: *Mycoplasma hominis*, *Bacteroides *and *Fusobacterium *species, *Streptococcus agalactiae *as well as *Gardnerella vaginalis*. When such organisms are found in the amniotic fluid of pregnant women prior to 20 weeks, the pregnancy usually ends 4 to 8 weeks later. It is also becoming increasingly evident that infection leading to preterm delivery actually occurs quite early in pregnancy and remains undetected for months prior to delivery [9]. It has been shown that *U. urelyticum *has been detected in some proportion of amniotic fluid samples at 15 to 18 weeks of gestation and majority of those women delivered around 24 weeks [10]. In another study interleukin-6 levels were high in the amniotic fluid at 15–20 weeks and spontaneous preterm delivery ensued as late as 32 to 34 weeks [9].

The release of endotoxins and exotoxins from bacterial infection leads to remodelling of the extracellular matrix (ECM) of gestational tissues (decidua, the cervix and the fetal membranes) which results in cervical ripening, fetal membrane rupture as well as placental and membrane separation from maternal tissues [11,12]. A number of secreted substances including Tumour Necrosis Factor-α (TNF-α), Interleukin-1α (IL-1α), IL-1β or IL-6 stimulate prostaglandin synthesis and release of metalloproteases. The former stimulate uterine contractions while the latter degrade the chorioamniotic membranes leading to rupture as well as remodel the collagen in the cervix and soften it [6].

## Reductionists versus systems approach in the studies on pathogenesis and early detection of preterm labor

As mentioned earlier, intraamniotic infection (IAI) is often chronic and usually asymptomatic until contractions begin or membranes prematurely rupture. It is therefore crucial to identify women at risk at early stages so that appropriate treatment strategies can be implemented.

Clinical medicine in general has been basically reductionist which can be best exemplified by the focus on a causative dominant factor. This for ages has resulted in the natural inclination to isolate the single factor that is most responsible for the given pathological status [13]. It has been widely accepted that every disease has its own unique target which would be most suitable for medical intervention. Since for infection the most obvious target is pathogen the methods for detection of subclinical infection leading to preterm labor have been in most of the cases concentrated on detecting bacteria or measuring different "source-related" compounds (eg. cytokines) in amniotic fluid, vaginal secretion or serum. As amniocentesis is not advocated in women who are in labor, alternative biological fluids, such as cervicovaginal secretions or serum are preferable as a source for diagnostic procedures.

There are many examples in general medicine, also in obstetrical diseases including preterm labor, where reductionism by means of focusing on a single factors and/or events is tremendously helpful and efficient as a way to study pathogenesis as well as search of new biomarkers.

It can among others be exemplified by the positive results on tests of vaginal secretions for bacterial vaginosis (defined as a decrease in the normally occurring *Lactobacillus *species and a massive increase in other organisms) which were found to be associated with intrauterine infection and were sufficient to predict preterm delivery [14]. It is widely accepted that chronic intrauterine infection leads to degradation of extracellular chorodecidual basement membrane, causing leakage of fibronectin (a protein of the placental membranes) into the vagina, which is the best predictor of chorioamnionitis and subsequent preterm delivery [15].

Although most of the studies showed that concentrations of high levels of many different cytokines, including TNF-α, IL-1, IL-6 or IL-8 are associated with preterm delivery [16,17], the results from two recent studies suggest that immune response is more complex and likely involves an interplay of different factors. In particular circumstances chronic immune status may lead to hyporesponsiveness, as represented by lower concentrations of some types of cytokines. In one of the studies it was reported that, among women with vaginal colonization by anaerobic Gram-negative rods or G. vaginalis at 18–22 weeks gestation, there exists an imbalance in the IL-1β and IL-1ra (receptor antagonists) immunological response. An elevated IL-1β concentration and a diminished IL-1ra:IL-1β ratio identified women at increased risk for spontaneous preterm delivery [18].

In another study Simhan *et al. *performed measurement of IL-1β, Il-6 and IL-8 in cervical fluid from 403 women at 8 to 20 weeks of gestation and found that genital tract immune hypo-responsiveness, as measured by decreased rather than increased lower genital tract concentrations of studied cytokines, is associated with subsequent chorioamnionitis [19]. The authors hypothesised that decreased concentrations of proinflammatory cytokines may represent diminished ability to appropriately respond to potential pathogens. It was also suggested that decreased cytokine concentrations reflect an inherent genetically regulated hyporesponse, which permits an infection to become chronic by allowing organisms to ascend into the uterus without causing a clinically-evident infection [19].

The above described successes of reductionist approach, which are based on a priori based hypotheses, leave less room for more contextual information. Novel types of "omics" methodologies that are based on platforms for simultaneous measurement of many different factors seem to effectively supersede hitherto applied one-by-one-factor reductionist approaches. It seems especially relevant to complex chronic diseases like preterm labor where the act of reduction (from larger to smaller) may result in the lose of attention to other primarily unexpected causative factors. These are probably more amenable to systems approach methodology which tends to generate hypotheses as a consequence of inherent technical structure of the assay [20].

**Figure 1:**
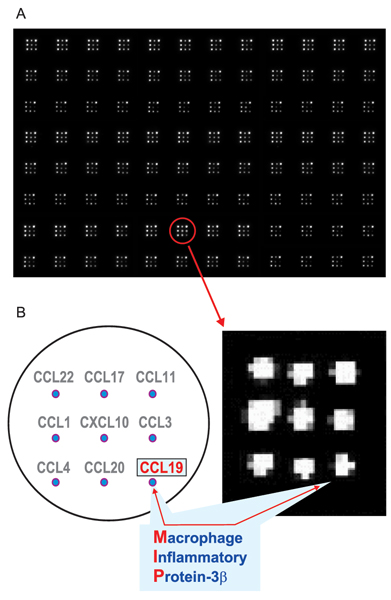
**Mini-array, sandwich-type, enzyme-linked immunosorbent assay for chemokine detection**. **(A) **Rows and columns in the image represent wells, of 96-well polystyrene plate, in the bottom of which monoclonal antibodies (mAbs) were spotted in a 3 × 3 pattern. After detection the plate was imaged with cooled charge-coupled device (CCD) camera. **(B) **Location of anti-chemokine mAb spots in the mini-array and CCD camera image of a 3 × 3 mini-array within a single well. Among nine of the studied chemokines it is CCL19 (also previously known as MIP-3β) which was significantly decreased in sera of patients with preterm labor (see the text for full details).

In our recent study we employed a novel proteomic mini-array assay to quantitate serum levels of different chemokines in laboring and non-laboring gravidas during preterm and term delivery (Figure 1). We excluded patients with clinical signs of infection, and found that among all of the studied chemokines it is only CCL19 (also previously known as Macrophage Inflammatory Protein-3β i.e. MIP-3β) of which levels were significantly lowered in women with preterm delivery. Taking into consideration the results from the two above-cited studies [18,19] we have also assumed that lower concentration of CCL19 might fit into the scenario of diminished immuno-responsiveness [21].

The proteomic method that was used in our work is actually just one of the many in the burgeoning field of genomics and proteomics. Although the results were obtained with relatively small amount of examined factors, nine altogether, we were able to pave the way for future confirmatory studies on the role of hitherto unexplored chemokine in the context of parturition. It therefore appears more and more evident that the genes, proteins or peptides that are preferentially expressed and identified in a disease or pathologic state are well suited for the development of much more robust, sensitive and specific diagnostic assays. For example, Gravett *et al. *[22] had for the first time used surface-enhanced laser desorption-ionization/time-of-flight (SELDI-TOF) mass spectrometry in order to characterize amniotic fluid peptides in *Rhesus *monkeys before and after experimental intra-amniotic infection (IAI). The resultant peptide profiles were subsequently verified in a cohort of women with preterm delivery. The objective of the study was to discover novel biomarkers for subclinical or occult IAI. It was found, that calgranulin B (expressed by macrophages and by epithelial cells in acutely inflamed tissues) and a unique restrictive fragment of Insulin-like Growth Factor Binding Protein 1 (IGFBP-1) – synthesized by both fetal membranes and maternal decidua, were differentially expressed in the amniotic fluid and sera during intra-amniotic infection prior to the appearance of clinical symptoms. In contrast, elevated IL-6 concentrations have not been reported in maternal serum in association with intra-amniotic infection. However, among all of the detected proteins those had been defensin-1 and defensin-2 which were subsequently, by the use of similar proteomic approaches, found to be of major importance in the inflammatory response to amniotic infection [23,24]. It was concluded that these diagnostic protein expression signatures might have application in the early detection of IAI [25].

With the advent of cDNA array technology, it has also become possible to examine simultaneously the expression of hundreds or thousands of genes encoding multiple classes of proteins, and hence to assess global changes in gene expression in response to a given condition e.g. in the myometrium in pregnancy and labor or inflammation leading to preterm labor [26,27]. In one of the first microarray studies on preterm delivery, Marvin *et al. *used cytokine-specific 384 gene array to compare expression level of term and preterm gestational membranes (with and without chorioamnionits) [28]. The study for the first time presented evidence for differences not only in the expression of some new inflammation-associated genes, including Macrophage Inflammatory Protein-1β (MIP-1β) and Pulmonary and Activation-Regulated Chemokine (PARC), but also noted a differential expression patterns of classes of genes, grouped according to function (e.g. chemokines) [28].

More recently, it was presented that there exists a decreased expression of proteinase inhibitor 3 (PI3) in the preterm premature rupture of membranes (pPROM) cases as shown by cDNA microarrays which contained 9128 sequence-verified elements that represented a total of 8502 unique clones [29]. Although PI3 (a low-molecular weight serine proteinase inhibitor that is capable of inhibiting neutrophil elastase) had not been implicated previously to play a role in parturition, the authors proposed that that the production of PI3 in the fetal membranes protects the tissue from damage that could be caused by increased activity and availability of neutrophil elastase. Patients who are not capable of producing adequate amounts of PI3 may be predisposed to PPROM. This provides a novel pathway for investigation in the pathobiologic condition of preterm birth that was obtained by a novel genomic approach which out of more than 8000 factors was efficient to point to one, potentially relevant [29].

## Conclusion

Regardless of which omics-based approach was used the conclusions from the above-cited studies, and our own, are quite similar and concern great potential utility of those methods to identify specific biomarkers and diagnostic profiles of preterm labor. Although "omics" methodologies require increasingly sophisticated computational and mathematical tools which are only beginning to unravel its full potential we believe the systems assumption that the forest (e.g. preterm labor) cannot be explained by studying the trees individually (one by one factor) will become validated [13]. Only by harnessing the "HuGe" (human genome) potential of the methods through creating collaborative networks based on interdisciplinary teams, as very recently proposed for the integration of genomics into obstetrics and gynecology [30], we can effectively increase our understanding of the particular aetiologies involved in this one of the most challenging obstetrical maladies.

## Competing interests

The authors declare that they have no competing interests.

## Authors' contributions

Authors have made equal contribution. All authors read and approved the final manuscript.
